# Defensive tolerance drives the reprogramming and dysfunction of infiltrating pathogenic B cells assuring the maintenance of tolerance

**DOI:** 10.21203/rs.3.rs-7236564/v1

**Published:** 2025-08-18

**Authors:** Koki Hayashi, Takahiro Yokose, Jenna Lancey, Edward S. Szuter, Brandon Kwon, Fabian Murillo, Alessia Giarraputo, Ivy Rosales, Wenjun Li, Michael T. Guinn, Grant Cosimi, Sarah Rose Odutola, Joylyn Kim, Peter T. Sage, Giuseppe Tarantino, Amy Huang, David Liu, Derek Effiom, Genevieve M. Boland, Sonia Cohen, Oliver McCallion, Fadi Issa, Collin Jordan, Xunrong Luo, Andrew S. Liss, David A. Ruddy, Michelle Piquet, Paul S. Russell, Robert B. Colvin, Joren C. Madsen, A. Benedict Cosimi, Daniel Kreisel, Alessandro Alessandrini

**Affiliations:** 1 Center for Transplantation Sciences, Department of Surgery, Massachusetts General Hospital and Harvard Medical School; Boston, MA, USA.; 2 Department of Surgery, Massachusetts General Hospital and Harvard Medical School; Boston, MA, USA.; 3 Department of Pathology, Massachusetts General Hospital; Boston, MA, USA.; 4 Departments of Surgery, Pathology, and Immunology, Washington University in St. Louis, St. Louis, MO,; 5 Michael E. DeBakey Department of Surgery, Baylor College of Medicine; Houston, TX, USA.; 6 Transplantation Research Center, Brigham and Women’s Hospital and Harvard Medical School; Boston, MA, USA.; 7 Department of Medical Oncology, Dana-Farber Cancer Institute, Harvard Medical School, Boston, MA, USA, and Broad Institute of MIT and Harvard, Cambridge, MA, USA; 8 Massachusetts General Hospital Cancer Center, Boston, MA, USA; 9 Nuffield Department of Surgical Sciences, University of Oxford, United Kingdom; 10 Department of Medicine, Duke University, Durham, North Carolina, USA; 11 Novartis Biomedical Research, Oncology, Cambridge, Massachusetts, USA; 12 Division of Cardiac Surgery, Massachusetts General Hospital; Boston, MA, USA.; 13 These authors contributed equally: Koki Hayashi, Takahiro Yokose, Jenna Lancey, Edward S. Szuter; 14 Corresponding and senior author.

## Abstract

We previously showed that infiltrating cytotoxic immune cells are reprogrammed to regulatory-like/exhausted cells within accepted kidney allografts through a ‘defensive tolerance’ mechanism. We observed a regulatory B cell (Breg) signature within the accepted kidney. Here we show that despite a Breg phenotype, neither B cell depletion nor the use of μMT recipients which lack B cells, resulted in kidney allograft rejection. Negative regulators of B cell function, *Siglecg* and *Fcgr2b*, show increased expression in both accepted kidney and lung allografts. Kidney allografts transplanted in B6.*Fcgr2b* KO recipients underwent antibody mediated rejection. Hypothesizing that similar mechanisms in a tumor microenvironment may attenuate anti-tumor immunity, we observed that expression of *SIGLEC10*, the human homolog of *Siglecg*, was associated with resistance to anti-PD1 therapy in human melanomas. In conclusion, B cell expression of FcγRIIB and Siglec-G appear to play an essential role in maintaining transplant tolerance and in tumor evasion of anti-tumor immunity.

A major goal in the field of transplantation is to identify and implement reliable protocols that induce allograft acceptance in the absence of ongoing immunosuppressive therapy^1–3^. This ‘holy grail’ would eliminate both the frequent development of chronic rejection and the debilitating side-effects of current standard-of-care treatment regimens^4,5^. To this end, we and others have studied how tolerance is induced and maintained in various natural biological scenarios, including pregnancy and tumors. Of unique value for defining specific mechanistic pathways are the spontaneous mouse transplant tolerance models, where kidney or liver allografts are accepted in certain strain combinations without the use of immunosuppression^6–8^. We have previously shown that accepted kidney allografts develop localized, intra-graft aggregates of various lymphocytes around the small vessels of the graft. We refer to these structures as regulatory Tertiary Lymphoid Organs (rTLOs), reminiscent of Tertiary Lymphoid Structures (TLS) found in solid tumors and tolerant lung allografts^6,7,9–13^. Immunohistochemistry showed that these rTLOs contain Foxp3^+^ regulatory T cells (Tregs), CD8^+^ and CD4^+^ T cells, B cells, conventional and plasmacytoid dendritic cells, and macrophages^6^. Serial analysis of single cell RNA sequencing (scRNAseq) data from CD45^+^ cells isolated from accepted kidney allografts revealed a shift from a T cell-dominant to a B cell-rich population by 6 months with an increased regulatory B cell signature^14^. These findings are consistent with B cell signatures that have been associated with renal transplant tolerance in humans^15–17^. The functionality of the B cell subsets identified in our kidney transplant model remains unclear. Therefore, the focus of this study was to assess the role that B cells play in kidney graft tolerance.

Here we show that neither depletion of B cells at different times post kidney transplant nor using μMT recipients that lack B cells resulted in rejection. Our scRNAseq data showed a temporal increase of *Fcgr2b* expression within the B cell clusters. *Fcgr2b* encodes for FcγRIIb, an inhibitory receptor on B cells, that regulates their function^18,19^. Using B6.*Fcgr2b* knockout (KO) recipients, we demonstrate that kidney allografts are rejected with a mean survival time of 21 days when compared to WT recipients. Histological analysis of the rejected kidneys from B6.*Fcgr2b* KO mice showed thrombotic microangiopathy, glomerulitis and capillaritis, and transplant glomerulopathy, features characteristic of acute and chronic antibody mediated rejection (AbMR).

Complementary to kidney transplantations, analysis of scRNAseq data from re-transplanted tolerized mouse lung allografts^20,21^ show that newly infiltrating B cells express *Siglecg* and *Fcgr2b* while resident B lymphocytes have a regulatory B cell (Breg) signature. Trajectory analysis shows that Breg-like cells arise from *Siglecg*+*Fcgr2b*+ B cells. The development of a Breg-like phenotype within a tolerized lung allograft is similar to our previous observations in a model of spontaneous kidney allograft acceptance^14^.

Lastly, in regard to malignancies, scRNAseq analyses of immune cells from the tolerized tumor microenvironment of spontaneous pancreatic tumors in the KrasLSL.G12D/+; p53R172H/+; PdxCretg/+ (KPC) mouse model for pancreatic ductal adenocarcinoma and human melanoma patients show a similar increased expression of *Siglecg* and *Fcgr2b* as well as *SIGLEC10 (*human homolog of *Siglecg*) and *FCGR2B* within the B cell population, respectively. Furthermore, increased *SIGLEC10* expression correlated with resistance to anti-PD1 therapy.

Our results suggest that B cells do not play a direct role in the induction and maintenance of kidney allograft tolerance. They rather infiltrate the kidney allografts as pathogenic cells and are reprogrammed to Breg/exhausted cells, first by the induction of *Siglecg* and *Fcgr2b*, followed by the increased expression of genes that define Bregs. This extends findings from our recent study that demonstrated that graft infiltrating alloreactive cytotoxic CD8+ T cells are reprogrammed within an accepted kidney allograft to an exhausted/regulatory-like phenotype mediated through a process we call ‘defensive tolerance’^22^. This intra-graft reprogramming appears to be a secondary, regulatory, and protective mechanism that is characteristic of tolerance, whether it be an accepted kidney or lung allograft in the absence of immunosuppression, or the tumor microenvironment. Overall, our findings further add to our understanding of allotransplant tolerance, with implications that can be expanded to how tumors escape the host’s immune system.

## RESULTS

### Depletion of B cells does not prevent acceptance of kidney allografts.

**Fig. 1.**
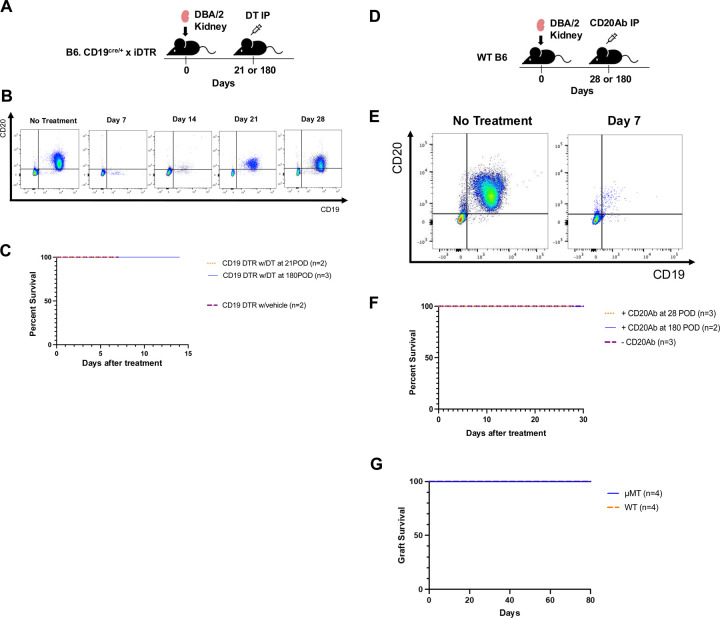
B cells are not required for the induction and maintenance of kidney allograft acceptance. (**A**) Schematic showing the experimental design for CD19^+^ cell depletion using B6. [CD19^Cre/+^iDTR^fl/+^] mice and intraperitoneal (IP) injection of diphtheria toxin (DT). (**B**) Serial flow cytometric analysis of CD19+ and CD20+ cells in peripheral blood mononuclear cells (PBMC) following DT administration. (**C**) Kidney allograft survival following DT injection at post-operative day (POD) 21 (n = 2) or POD 180 (n = 3), compared to vehicle-treated controls (n = 2). (**D**) Schematic of the experimental design for CD20^+^ cells depletion using IP injection of anti-CD20 antibody (CD20Ab) into B6 wildtype (WT) recipients of DBA/2 kidneys. (**E**) Serial flow cytometric analysis of CD19+ and CD20+ cells in PBMC over time following CD20Ab administration. (**F**) Kidney allograft survival following CD20Ab injection at POD 28 (n = 3) or POD 180 (n = 2), compared to untreated controls (n = 3). (**G**) DBA/2 kidney allograft survival in B6.μMT recipients (n=4), which lack mature B cells, compared to B6 WT recipients (n = 4).

We previously described a temporal increase in a Breg-like phenotype within spontaneously accepted kidney allografts^14^. To ascertain the significance of this observation and assess the functionality of B cells in the maintenance of renal allotransplant tolerance, we utilized B6.[CD19^Cre/+^iDTR^fl/+^] mice as recipients, which express the diphtheria toxin receptor (DTR) on B cells and therefore allow for the depletion of CD19-expressing B cells after systemic treatment with diphtheria toxin (DT)^23^. Kidney allograft recipients were DT-treated at 28- or 180-days post-transplant (Fig. 1A). Flow cytometric analysis of peripheral blood mononuclear cells (PBMC) showed that B cells were depleted for up to 14 days before they began to recover 21 days post-treatment (Fig. 1B). Unlike the depletion of recipient regulatory T cells (Tregs) that results in the rejection of the DBA/2 kidney allografts 6–9 days post-DT treatment of B6.Foxp3-DTR recipients^6^, depletion of B cells did not result in the loss of the kidney graft, and maintained a 100% survival rate, comparable to vehicle treated recipients (Fig.1C). We also depleted B cells in B6 WT recipients using anti-CD20 antibodies (Fig.1D), a regimen that results in the depletion of B cells for up to 7days (Fig. 1E). Similar to DT-treated B6.[CD19^Cre/+^iDTR^fl/+^] recipients, we observed no rejection of DBA/2 kidney allografts following anti-CD20 mediated B cell depletion (Fig.1F). Furthermore, kidney allografts are spontaneously accepted in B6.μMT recipients with no rejection episodes up to 80 days post-transplantation indicating that B cells are not required for the induction or maintenance of tolerance in this model (Fig. 1G).

### Increased intra-graft expression of *Fcgr2b* and *Siglecg* within the B cell cluster.

**Fig. 2.**
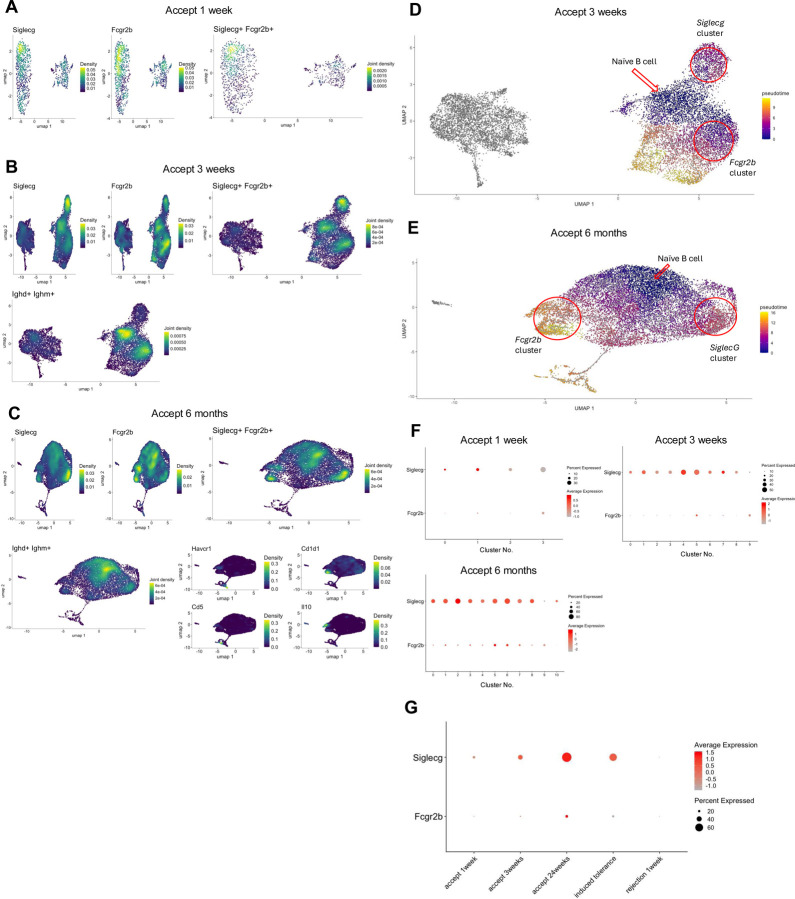
Temporal expression analysis of *Fcgr2b* and *Siglecg* in accepted and rejecting kidney allografts. (**A**) Density plots of *Siglecg*^+^*, Fcgr2b*^+^, and *Siglecg*^+^*Fcgr2b*^+^ cells within B cell clusters in accepted kidney allografts at 1-week post-transplantation. (**B**) Density plots of *Siglecg*^+^*, Fcgr2b*^+^*, Siglecg*^+^*Fcgr2b*^+^, and *Ighd*^+^*Ighm*^+^ cells in B cell clusters at 3 weeks post-transplantation. (**C**) Density plots show expression of *Siglecg*^+^*, Fcgr2b*^+^*, Siglecg*^+^*Fcgr2b*^+^*, Ighd*^+^*Ighm*^+^, and Breg markers, including *Havcr1, Cd1d1, Cd5, and IL10* at 3 weeks post-transplantation. (**D, E**) Pseudotime analysis of B cell clusters at 3weeks (**D**) and 6 months (**E**) post-transplantation. The red arrow marks the origin point representing naïve B cells, and the red circle highlights a cluster expressing *Siglecg* or *Fcgr2b*. Pseudotime graphic is overlaid on the UMAP plot on a gradient color scale. (**F**) Dot plots show progressive upregulation of *Siglecg* and *Fcgr2b* over time (1 week, 3 weeks, and 6 months post-transplantation) within B cell clusters. (**G**) Dot plot analysis showing higher expression of *Siglecg* and *Fcgr2b* in the spontaneous acceptance model (accept 1, 3, 24 weeks) and in an induced kidney acceptance model (where recipients are infused with donor splenocytes treated with the chemical cross-linker ethylenecarbodiimide (ECDI-SP)), when compared to a kidney allograft rejection model (B6 to DBA/2).

While our data suggest that B cells may not be involved in the induction and maintenance of kidney allograft tolerance, we have previously shown that *Siglecg* expression is temporarily increased within the B cell cluster in accepted kidney grafts^14^. *Siglecg* encodes for Siglec-G, which when expressed on B cells prevents the secretion of antibodies. It can also interact with CD22 on other cell types and induce the upregulation of Siglec-G on their surface^24–26^. We also showed that infiltrating cytotoxic CD8^+^ T cells are reprogrammed to a regulatory-like/exhausted phenotype within the tolerized kidney allograft accompanied by an increase in their *Fgl2* expression^22^. *Fgl2* encodes fibrinogen-like protein 2 (FGL2), which can be secreted by CD8+ Tregs and functions to induce Bregs and inhibit dendritic cell maturation by interacting with its receptor, FcγRIIB^27,28^. We next investigated the expression of *Fcgr2b* (gene encoding FcγRIIB) as an additional Breg marker in the DBA/2 to B6 spontaneous kidney allograft acceptance model. Concurrently, the expression of *Siglecg* was reassessed to provide a comparative analysis of the expression patterns of regulatory markers. We observed that *Fcgr2b* and *Siglecg* share analogous expression patterns within B cell and monocyte populations (Supplemental Fig. 1), with similar levels of expression density in corresponding areas of the B cell cluster (Fig. 2A). At one-week post-transplantation, both markers demonstrated a peak in expression concentration on overlapping subclusters within the B cell population (Fig. 2A). By three weeks post-transplantation, there is a noticeable concentration of expression in analogous regions of the B cell subcluster for both markers. Intriguingly, the peaks occur in complementary regions, with *Fcgr2b* being more pronounced in the central areas of the right cluster, while *Siglecg* shows higher intensity in the upper portion of the right cluster (Fig. 2B). To investigate the possibility of intra-graft reprogramming of naïve B cells to *Siglecg*^+^ or *Fcgr2b*^+^ B cells, we performed trajectory analysis of the scRNAseq data using Monocle3^29–31^. Density plots show that the central region of the right cluster is associated with naïve B cells (Fig. 2B). At six months post-transplantation, *Fcgr2b*’s expression overlaps with the zones of known Breg markers, such as *Havcr1*, *Cd1d1*, *Cd5*, and *Il10* (Fig. 2C). Trajectory analysis suggested that both *Fcgr2b*^+^ and *Siglecg*^+^ phenotypes mature from naïve B cells (Fig. 2D), and by 6 months post-transplantation, the *Fcgr2b*^+^ phenotype occurs later than *Siglecg* (Fig. 2E). The corresponding dot plots quantify these observations. At one-week post-transplantation, both *Siglecg* and *Fcgr2b* are expressed in a smaller subset of cells, with *Fcgr2b* demonstrating relatively low expression levels within subclusters 0 and 1 (Fig. 2F). At three weeks post-acceptance, *Fcgr2b* continues to exhibit low expression levels throughout the cluster (Fig. 2F). However, by six months post-transplantation, there is a noticeable increase in the average expression of *Fcgr2b* within the B cell cluster (Fig. 2F). This expression pattern raises the possibility that FcγRIIB plays a role in the long-term maintenance of tolerance.

To determine whether similar results could be observed in other mouse renal tolerance models, we evaluated the expression of *Fcgr2b* and *Siglecg* in an induced tolerance model involving BALB/c to B6 kidney transplantation. In this model, donor-specific tolerance is induced using a well-established regimen involving peri-transplant recipient infusions of donor splenocytes treated with the chemical cross-linker ethylenecarbodiimide (ECDI-SP)^32^. Dot plot analyses compared *Fcgr2b* and *Siglecg* expression across the induced tolerance model (day 15 post-transplantation), our spontaneous DBA/2 to B6 acceptance model (at 1 week, 3 weeks, and 6 months post-transplantation), and a rejection model (B6 to DBA/2) (Fig.2G). In the induced tolerance model, *Siglecg* was strongly expressed. The percentage expression of *Fcgr2b* was higher than in the rejection model. Thus, *Siglecg* and *Fcgr2b* display similar expression patterns within tolerant grafts across several models.

### Early intra-graft expression of *Fcgr2b* and *Siglecg* within the B cell cluster in retransplanted tolerant lung allografts.

**Fig. 3.**
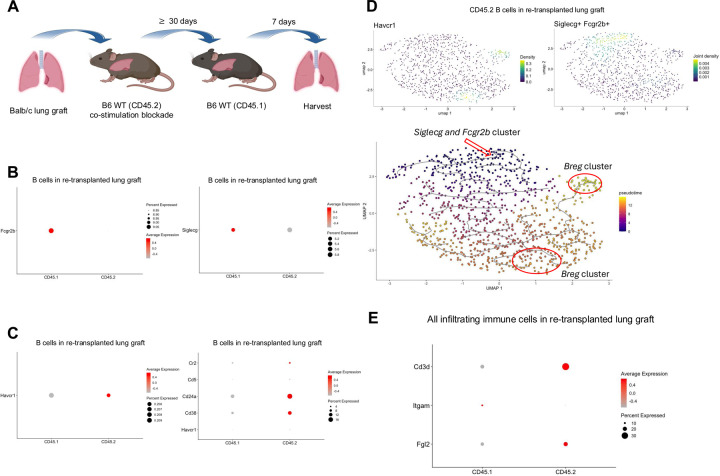
Temporal expression of *Fcgr2b*, *Siglecg*, and Breg markers in accepted lung allografts. (**A**) Schematic showing transplantation of Balb/c lungs into B6 WT recipients (CD45.2), treated with peri-operative co-stimulatory blockade, followed by re-transplantation into non-immunosuppressed B6 WT (CD45.1) recipients ≥30 days later. Graphic created with BioRender. (**B**) Dot plots of *Fcgr2b* and *Siglecg* expression in the CD45.1^+^ and CD45.2^+^ B cell clusters. (**C**) Dot plot analyses of Breg markers - *Havcr1, Cr2, Cd5, Cd24a, Cd38* - within the CD45.1+ and CD45.2^+^ B cell clusters and *Siglecg* expression in the CD45.1^+^ and CD45.2^+^ B cell clusters. (**D**) Pseudotime analysis of B cell clusters at seven days after re-transplantation. The red arrow marks the origin point representing *Siglecg*^+^*Fcgr2b*^+^ B cells, and the red circle highlights clusters expressing the Breg marker *Havcr1*. Pseudotime graphic is overlaid on the UMAP plot on a gradient color scale. (**E**) Dot plots show expression of *Fgl2* within the T cell cluster.

We have shown that peri-operative co-stimulation blockade results in the induction of tolerance after allogeneic lung transplantation in the mouse, which is associated with the development of tertiary lymphoid organs that are enriched in Tregs and also contain abundant B cells^20,21^. We have also reported that tolerance is maintained after re-transplantation of tolerant lung allografts into non-immunosuppressed secondary hosts. This model affords us the possibility of assessing and characterizing newly infiltrating immune cells and to compare them with immune cells that reside within the tolerant graft at the time of re-transplantation (Fig. 3A). Analysis of scRNAseq data of intra-graft immune cells show that resident B cells (CD45.2^+^) express lower levels of *Siglecg* and *Fcgr2b* when compared to newly infiltrating B cells (CD45.1^+^) one week after re-transplantation (Fig.3B). Interestingly, resident B cells have a Breg signature, including expression of *Cr2*, *Cd5*, *Cd24a*, *Cd38*, and *Havcr1* not seen in newly infiltrating B cells (Fig. 3C). Trajectory analysis of the CD45.2^+^ scRNAseq data showed that *Havcr1* arose from *Siglecg*^+^*Fcgr2b*^+^ B cells (Fig. 3D). These findings raise the possibility that *Siglecg* and *Fcgr2b*, expressed early within infiltrating B cells, may render them susceptible to reprogramming into Breg-like cells in response to Fgl2. Consistent with our published scRNAseq data from accepted kidney grafts, *Fgl2* is expressed in immune cells that reside in accepted lung grafts, specifically within the resident T cell population (Fig. 3E).

### Antibody-mediated rejection of kidney allografts in B6.*Fcgr2b* KO recipients.

**Fig. 4:**
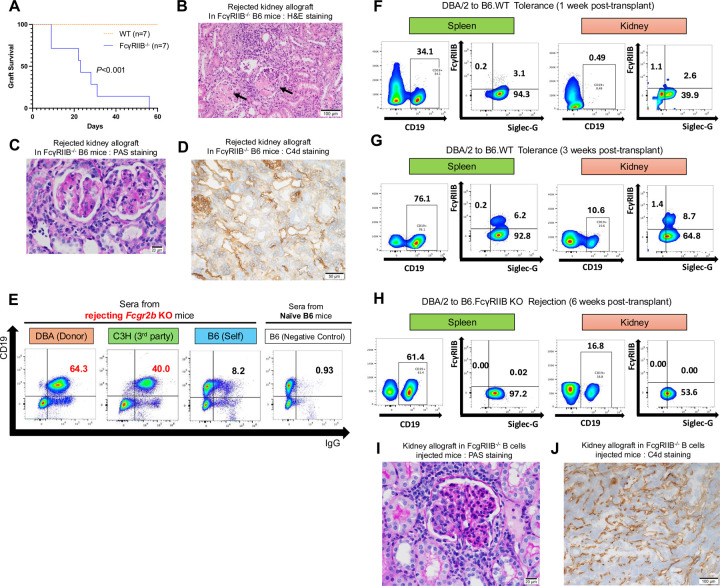
Antibody-mediated rejection of kidney allografts in FcγRIIB^−/−^ recipients. (**A**) DBA/2 kidney allograft survival in FcγRIIB^−/−^ recipients (n=13), compared to wild-type (WT) recipients (n = 13; *P*<0.001). (**B-D**) Histopathological analysis of rejected kidney allografts obtained from FcγRIIB −/− recipients. (**B**) H&E staining shows thrombotic microangiopathy. Arrows indicate mononuclear cell infiltration. Day 35. Scale bars: 100μm. (**C**) PAS staining shows glomerulitis and capillaritis. Day 35. Scale bars: 20μm. (**D**) Immunohistchemistory shows increased C4d deposition in FcγRIIB −/− kidney allograft. Day 39. Scale bars: 50μm. (**E**) Flow cytometric analysis of serum antibodies reactive to mouse spleen cells. Sera from FcγRIIB −/− recipients exhibited higher antibody reactivity against DBA/2 (donor, 64.3%) and C3H (third-party, 40.0%) cells, compared to B6 (self, 8.2%) and negative control (0.93%). (**F-H**) Flow cytometric analysis of SiglecG^+^ FcγRIIB^+^ cells within the CD19^+^ B cell population isolated from spleens and kidney allografts at 1 week (**F**) and 3 weeks (**G**) post-transplantation in the tolerance model and 6 weeks post-transplantation in the FcγRIIB −/− rejection model (DBA/2 to B6 FcγRIIB^−/−^) (**H**). The data in (**F-H**) are representative of triplicate experiments. (**I, J**) Histopathological analysis of kidney allografts obtained from FcγRIIB −/− B cells injected recipients 4 weeks post-transplantation (n=3). (**I**) PAS staining shows glomerulitis. Scale bars: 20μm. (**J**) Immunohistchemistory shows increased C4d deposition in FcγRIIB −/− B cells injected kidney allograft. Scale bars: 100μm.

Increased expression levels of *Fcgr2b* and *Siglecg* in B cells within accepted kidney allografts, combined with an absence of circulating donor-specific antibodies (DSAs), raises the possibility of a suppressive mechanism against antibody production. To assess if *Fcgr2b* affects the induction kidney allograft tolerance, we performed survival and immunohistochemical analysis using gene-deficient recipients. The survival of DBA/2 kidneys is significantly shorter after transplantation into B6 FcγRIIB^−/−^ (n=13; MST=31 days) compared to B6 WT mice (n=13) (Fig. 4A). After transplantation into FcγRIIB^−/−^ recipients, kidney allografts exhibit thrombotic microangiopathy, as highlighted by the mononuclear cell infiltration, glomerulitis, and capillaritis (Fig. 4B, C). These pathological changes are accompanied by an increase in circulating DSAs and enhanced C4d deposition (Fig. 4D, E). Collectively, these findings are indicative of antibody-mediated rejection when recipients lack FcγRIIB.

We observed a temporal increase in FcγRIIB^+^Siglec-G^+^ and overall Siglec-G^+^ B cells in the recipient spleen and kidney allograft after transplantation into WT recipients (Fig. 4F, G). Analysis of splenic and intra-graft B cells from FcγRIIB^−/−^ recipients at the time of rejection shows a similar abundance of Siglec-G^+^ B cells (Fig. 4H) suggesting that Siglec-G expression on B cells is not sufficient to prevent antibody-mediated rejection observed in FcγRIIB^−/−^ recipients.

Analysis of our scRNAseq dataset shows that *Fcgr2b* is also expressed in the myeloid clusters in addition to the B cell cluster (Supplemental Fig. 1). To confirm that FcγRIIB^−/−^ B cells can directly result in the induction of rejection in kidney allografts in our model, we adoptively transferred 2×10^6^ purified FcγRIIB^−/−^ B cells (Supplemental Fig. 2A, B) into WT B6 mice prior to transplanting a DBA/2 kidney. Analysis of the kidney allograft at 4 weeks post-adoptive transfer and post-transplantation shows signs of glomerulitis (Fig. 4I) and C4d deposition (Fig. 4K), as well as an increase in circulating DSA (Supplemental Fig. 2C).

### The B cell population in tumors exhibits increased expression of *Fcgr2b* and *Siglecg*.

**Fig. 5.**
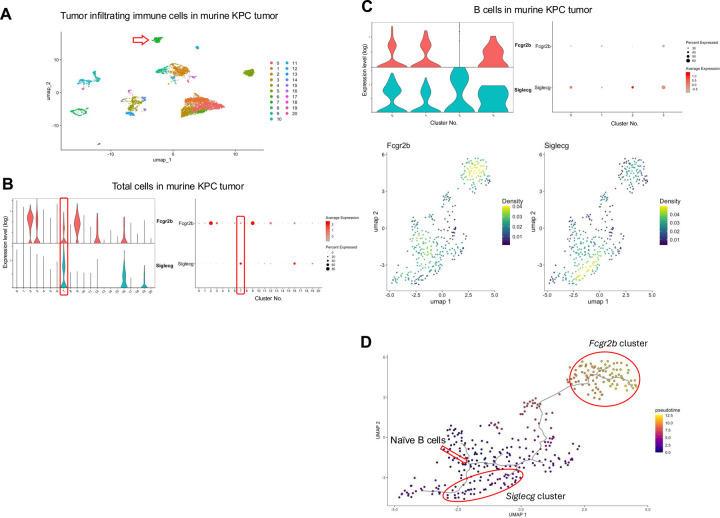
Expression of *Fcgr2b* and *Siglecg* within the B cell cluster in murine KPC tumors. (**A**) UMAP plot of all cells isolated from murine KPC tumors. The **red arrow** indicates the B cell cluster. (**B**) Violin and dot plots analysis of *Fcgr2b* and *Siglecg* expression in total cells. The B cell cluster is boxed in red. (**C**) Violin, dot and density plots of *Fcgr2b* and *Siglecg* expression in the B cell cluster. (**D**) Pseudotime analysis of the B cell cluster in murine KPC tumors. The red arrow indicates the origin point representing naïve B cells, and the red circle highlights a subpopulation expressing *Fcgr2b* and *Siglecg*. Pseudotime graphic is overlaid on the UMAP plot on a gradient color scale.

While accepted kidney and lung allografts develop regulatory tertiary lymphoid organs (rTLOs), which are thought to contribute to the maintenance of graft tolerance, the tumor immune microenvironment similarly forms tertiary lymphoid structures (TLSs) that resembles rTLOs in our transplant models^6,7,9–13^. These TLSs contain B and T cells^33^ and may play a role in sustaining tumor immune tolerance. We have previously shown that cytotoxic CD8^+^ T cells are reprogrammed to regulatory-like/exhausted cells in our kidney allograft spontaneous acceptance model and similar regulatory-like/exhausted CD8^+^ T cells were present in tumors, including KPC tumors^22^. Given these similarities, we examined whether *Siglecg* and *Fcgr2b* are also highly expressed within the B cell population in tumors. Figure 5 summarizes the scRNAseq data from spontaneous pancreatic tumors in the KPC mouse model. Analysis of total cells revealed elevated expression of *Fcgr2b* and *Siglecg* in the B cell cluster (Fig. 5A, B). Sub-clustering of B cells further confirmed that both genes are upregulated in a pattern like that observed in tolerant allografts (Fig. 5C). Density plots for each marker show that B cells expressing *Fcgr2b* or *Siglecg* are enriched in distinct subclusters (Fig. 5C). Additionally, trajectory analysis of the scRNAseq data via Monocle3^29–31^ shows a cellular transition within this cluster from naïve B cells to *Siglecg*- and then over time to *Fcgr2b*-dominated expression (Fig. 5D). This is also demonstrated within the density plot of each respective marker showing that the greatest density of B cells that expresses *Fcgr2b* or *Siglecg* is represented by different B cell subclusters (Fig. 5C). These findings raise the possibility of a similar mechanism of immunomodulation taking place within B cells in pancreatic tumors and accepted kidney allografts.

### Increased *SIGLEC10* gene expression within the B cell population in human melanomas correlates with poor response to anti-PD1 therapy.

**Fig. 6.**
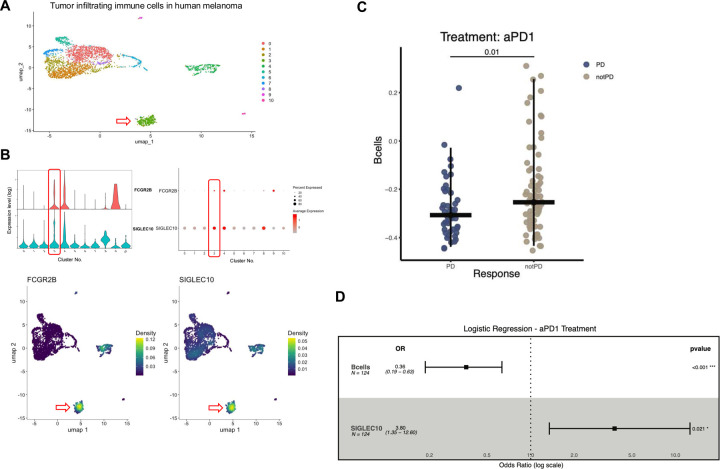
Expression of *FCGR2B* and *SIGLEC10* within the B cell cluster in human melanomas. (**A**) UMAP plot of tumor-infiltrating immune cells isolated from human melanomas. The red arrow indicates the B cell cluster. (**B**) Violin, dot and density plots show *FCGR2B* and *SIGLEC10* expression within the B cell cluster among all tumor-infiltrating immune cells. The B cell cluster is boxed in red, and the red arrow indicates its location. (**C**) Boxplot comparing the B cell normalized enrichment score estimated via ssGSEA in patients treated with anti-PD-1 therapy(aPD1), stratified by clinical response (progressive disease [PD] vs. non-progressive disease [notPD]). The B cell signature includes the following genes: *BLK, CD19, MS4A1, TNFRSF17, FCRL2, FAM30A, PNOC, SPIB, and TCL1A*. (**D**) Forest plot showing odds ratios (OR) and 95% confidence intervals (CI) for the association between B cell abundance and *SIGLEC10* expression with response to anti-PD1 therapy. ORs are shown on a log scale, with a dotted vertical line indicating the null value (OR = 1). Each square represents the estimated OR for one predictor, and horizontal lines denote the 95% CI. Values were standardized (z-score) prior to model fitting to allow interpretation per 1 standard deviation increase (n=124). Statistically significant associations (p < 0.05) are indicated with asterisks.

To determine whether B cell signature similar to the one observed in murine tumors and tolerant allografts is present in human cancers, we analyzed publicly available scRNAseq data from human melanoma samples^34^, focusing on the expression of *FCGR2B* and *SIGLEC10* (the Siglec-G counterpart in humans) in the B cell compartment. Examination of tumor-infiltrating immune cells revealed that *SIGLEC10* and *FCGR2B* are frequently and highly expressed within the B cell cluster (Figure 6A, B). While TLSs may play a role in sustaining tumor immune tolerance, recent data in melanoma demonstrated that TLSs may contribute to the maintenance of anti-tumor immunity, with higher expression of a TLS gene signature associated with greater clinical benefit observed in patients treated with immunotherapy^10^. First, we investigated whether SIGLEC10 and FCGR2B are highly expressed within the B cell population in human melanomas. Bulk RNA-sequencing^35^ was performed on resected tumors from patients with metastatic melanoma (n=124). All patients received anti-programmed cell death protein-1 (aPD1) immunotherapy with subsequent clinical follow up. Analysis of CD45^+^ cells revealed that SIGLEC10- and FCGR2B-expressing cells were densely located within the B cell populations, in addition to macrophage clusters (Fig. 6B). Patient clinical response data were aggregated into progressive disease (PD) and not-progressive disease (notPD). Single-sample gene-set enrichment (ssGSEA) analyses demonstrated enrichment of a B cell signature in notPD tumors (p=0.01) which were effectively patients who responded to aPD1 (Fig. 6C). A multivariate logistic regression analysis further revealed that high expression of *SIGLEC10*, when evaluated in combination with the B cell signature, was significantly associated with poor clinical response and disease progression (OR 3.80, 95% CI 1,35 – 12.60; p =0.021) (Fig. 6D). These results suggest a potential immunosuppressive role for *SIGLEC10* in the context of anti-PD1 resistance.

## DISCUSSION

Kidney allografts are spontaneously accepted without the use of immunosuppression in certain murine strain combinations^6–8,36,37^. The accepted allografts are characterized by the presence of rTLOs that form perivascularly and contain various immune cell types^6,7,14^. We have shown through both bulk mRNA and scRNAseq analyses that CD8^+^ T cells were the abundant intra-graft cell type at 1 and 3 weeks post-transplantation, and observed a shift to a B cell signature by 6 months post-transplantation^7,14^. Additionally, the B cell signature is reflective of a regulatory one, where we observed an increased expression of Breg markers, both at the transcriptional and protein level^14^. These findings were corroborated by flow cytometric and immunohistological analyses^7,14^. Recently, we showed that graft-infiltrating CD8^+^ T cells expressed cytotoxic markers (*Gzmb* and *Ifng*) as early as 1 week after transplantation and by 3 weeks were reprogrammed into exhausted/regulatory-like cells expressing *Fgl2*, *Il2rb*, *Pdcd1*, *Tox*, and *Lag3* through a process we have termed ‘defensive tolerance.’^22^ In this study we focused on examining the B cell population within the accepted kidney allografts in our transplant model.

We have shown that depletion of B cells, either pharmacologically or genetically, at early and late times post-transplantation did not result renal graft rejection, suggesting that Breg-like cells are not needed for the maintenance of tolerance in our spontaneous kidney acceptance model. This is contrary to our previous observations with Treg depletion in this model^6^. We further show that spontaneous acceptance of kidney allografts occurs in B6.μMT recipients indicating that B cells are not required for the induction of tolerance.

We have previously shown that one of the genes that was highly expressed within the graft at 3 weeks post-transplantation within the CD8^+^ T cell cluster is *Fgl2*^22^, which encodes for Fibrinogen-like protein 2 (Fgl2). It has been shown that regulatory CD8^+^ T cells secrete Fgl2, which is known to be immunomodulatory^12,27^. The receptor for Fgl2 is FcγRIIB^18,38^. In humans and mice, FcγRIIB, also known as CD32B, is the only inhibitory receptor of the Fcγ receptor family. FcγRIIB is encoded by *Fcgr2b*, and is present on the surface of B cells, dendritic cells, and myeloid effector cells^18,38^. FcγRIIB can mediate humoral immunity by heightening the BCR activation threshold and inhibiting B cell antigen presentation to helper T cells. FcγRIIB also directly alters antigen presentation by inhibiting DC maturation^18^. Of relevance, we found a temporal increase in *Fcgr2b* expression within the B cell clusters in accepted kidney allografts. This increase in expression mirrored a similar pattern in *Siglecg*^14^, which encodes for Siglec-G, a molecule that when expressed on B cells prevents the secretion of antibodies. Its interaction with CD22 on other cell types induces the upregulation of Siglec-G on their surface^24–26^. Flow cytometry data show that by 3 weeks, Siglec-G^+^ B cells also co-express surface FcγRIIB, both in the graft and in the recipient spleen. To ascertain the importance of FcγRIIB, we transplanted DBA/2 kidneys into B6.FcγRIIB^−/−^ recipients, where we observed antibody-mediated rejection with an increase in circulating DSAs and C4d deposition in the graft. DSA is never observed in our spontaneous acceptance model^6^. Adoptive transfer of purified FcγRIIB^−/−^ B cells into WT recipients confirmed the role of B cells in the induction of AMR in kidney allograft recipients. These data suggest that not only are B cells not needed for the induction and maintenance of kidney graft acceptance, but their reprogramming from pathogenic B cells to Breg-like cells in the tolerant graft may be a protective mechanism, reminiscent of our observations with graft-infiltrating cytotoxic CD8^+^ T cells^22^. Interestingly, in the lung retransplant model, scRNAseq analysis of resident and newly infiltrating B cells showed a Breg signature in the resident B cell population with low expression of *Fcgr2b* and *Siglecg*, while newly infiltrating B cells expressed both *Fcgr2b* and *Siglecg* with little or no expression of Breg genes. This is suggestive of a mechanism where *Fcgr2b* and *Siglecg* are upregulated in newly infiltrating B cells within the tolerant microenvironment, are eventually reprogrammed to Breg-like cells, and maintained as innocuous rather than their original pathogenic state.

We have previously shown that graft-infiltrating cytotoxic CD8^+^ T cells are reprogrammed to regulatory-like cells, including *Cd8*^+^*Fgl2*^+^*Il2rb*^+^ cells^22^. Similar cell populations were also observed in spontaneous murine tumor models^22^. We hypothesized that in the models of both spontaneous acceptance of kidney allografts and spontaneous murine tumors, infiltrating pro-inflammatory cells, such as cytotoxic CD8^+^ T cells, are reprogrammed to regulatory-like/exhausted cells because of a pro-tolerance microenvironment through a process we called ’defensive tolerance’^22^. In this study, using a scRNAseq cohort from the KPC mouse tumor model that we used in our previous study where we looked at reprogramming of cytotoxic CD8+ T cells^22^, we observed a gene expression pattern of *Fcgr2b* and *Siglecg* within the B cell population of murine pancreatic tumors that resembled our findings in spontaneously accepted kidney allografts. We observed similar increased expression of *FCGR2B* and *SIGLEC10* (the human homolog of *Siglecg*) in the B cell cluster from human melanomas^34,39^. Interestingly, we also observed a correlation between anti-PD1 therapy resistance and progression of disease in human melanoma patients that exhibited increased expression of *SIGLEC10*. The expression of these negative regulators of B cell function and their association with tumor growth and survival is not unprecedented. For example, studies have shown that expression of *FCGR2B* and *SIGLEC10* in gliomas was higher than in normal tissue where it was associated with a poor prognosis^40,41^. High expression of Siglec-10 on tumor-associated macrophages in stage 1 lung adenocarcinoma along with expression of tumor CD24 was associated with increased risk of recurrence^42^, and the Siglec-10-CD24 axis inhibits the phagocytosis of tumor cells by macrophages^43^. Siglec-10^hi^ tumor-associated macrophages are also associated with poor prognosis in patients with hepatocellular carcinoma^44^.

In summary, and as we previously showed with infiltrating cytotoxic CD8^+^ T cells^22^, pathogenic B cells are reprogrammed to a regulatory-like and innocuous phenotype within tolerant allografts through a process we call ‘defensive tolerance’. What drives the expression of *Fcgr2b* and *Siglecg* in B cells within the accepted kidney and in tumors has yet to be determined and will be the focus of future studies. Our further understanding of how this reprogramming is accomplished in tolerant allografts or tumors has implications for the design of novel therapeutics that will be more effective and clinically applicable for achieving allotransplant tolerance and for the efficacious anti-tumor immunity.

## ONLINE METHODS

### Sex as a biological variable

Our study exclusively examined male mice. We have done some kidney allotransplants using female recipients and have obtained similar results with regards to the induction of tolerance but there was greater variability regarding survival rates, not due to rejection of the renal allograft, but from technical complications resulting from our DBA/2 to B6 kidney transplantation technique when using female recipients.

### Mice

The C57BL/6J (B6, H2^b^), DBA/2J (DBA, H2^d^), BALB/c (H2^d^), B6.[CD19^Cre/+^iDTR^fl/+^] mice, Ptf1a^tm2(cre/ESR1)Cvw^/J, B6.129P2-Trp53^tm1Brn^/J, B6.129S4-Kras^tm4Tyj^/J, B6.129S4-Kras^tm4Tyj^/J, and B6.*Ptprc*^a^ *Pepc*^b^/BoyJ (CD45.1) mouse strains were purchased from Jackson Laboratories (Bar Harbor, ME). The B6.129S4-*Fcgr2btm1TtK* N12 (FcgR2b KO) mice were ordered from Taconic Biosciences.

All mice were held throughout the experiment under pathogen-free conditions in filter-top cages, containing an automatic water system. They were cared for according to methods approved by the American Association for the Accreditation of Laboratory Animal Care.

### Kidney transplantation

Kidney transplantation was performed as detailed previously^37^. In brief, a cuff of the aorta and inferior vena cava were anastomosed in an end-to-side manner. The ureter was anastomosed to the urinary bladder. A bilateral nephrectomy was also simultaneously performed.

### Lung transplantation

Lungs were transplanted from BALB/c (CD45.2) mice into C57BL/6 CD45.2 (B6) recipients that received treatment with peri-operative co-stimulatory blockade (anti-CD40 ligand (250 μg intraperitoneally (i.p.)) and CTLA4-Ig (200 μg i.p.) on post-operative days 0 and 2, respectively (BioXCell)^21^. At least 30 days after the primary transplant, these lungs were re-transplanted into non-immunosuppressed B6 CD45.1 mice, as previously described^11^. Seven days after re-transplantation, lung grafts were harvested, the tissue was digested, and single-cell suspensions prepared, as previously described^45^. Cells, pooled from two re-transplant recipients, were stained using antibodies for CD45.1, CD45.2 and 4’,6-diamidino-2-phenylindole (DAPI) (BD Biosciences) and were sorted by flow cytometry into DAPI^−^CD45.1^+^CD45.2^−^ and DAPI^−^CD45.1^−^CD45.2^+^ cells. Collected cells were centrifuged (300 relative centrifugal force for 5 minutes at 4 °C) and resuspended in collection buffer to a target concentration of 1,000 cells/μL. Cells were counted on a hemocytometer before proceeding. Single-cell suspensions were submitted to the Genome Technology Access Center core facility (Washington University) for single-cell genome-scale metabolic model (GEM) construction and complementary DNA synthesis and library construction. Samples were processed using the Chromium Single Cell 3′ Library & Gel Bead Kit (10X Genomics, v3) following manufacturer’s protocols. The libraries were sequenced on NovaSeq S4 (Illumina) targeting 50,000 reads per cell and 500 million read pairs per library. Cells were aligned to the mouse mm10–2020-A transcriptome using CellRanger (10x Genomics, v6.1.1) to generate feature-barcoded count matrices.

### Histological and Immunopathological Analysis

Sagittal sections of allografts were fixed in formalin, and sections were stained for hematoxylin and eosin (H&E) and periodic acid Schiff (PAS). Immunohistochemistry on frozen tissue was done using a rat monoclonal antibody to C4/C4d (16D2, Abcam). Pathologic evaluation was done using an Olympus BX53 microscope (Olympus) equipped with a digital camera (DP76, Olympus).

### Isolation of kidney and spleen cells

Prior to tissue collection, the kidney was injected and perfused with a collagenase solution (2ml of 1x HBSS, 1mL of Collagenase A, and 3uL of DNase I). The kidney was removed from the mouse, manually ground down, and digested in the same collagenase solution. The remaining undigested tissue was manually ground down using a 70-um strainer, washed 3 times, and resuspended using FACs buffer. The spleen was collected in RPMI and then manually ground down using an insulin syringe plunger over a 70um strainer. 1 mL of ACK Lysis Buffer (Gibco – ThermoFisher Scientific) was added to remove red blood cells from the spleen. Cells were washed three times and resuspended in FACs Buffer.

### Flow cytometry analysis

Cells were plated and stained with viability dye (eFlour 506, 1:1000 dilution, Invitrogen, L34976 A), and incubated in darkness for 30 minutes. After viability staining, CD16/32 Fc Block (1:100 dilution, BioLegend, 101302) was added to each sample for a 5–10 minutes. Cells were then washed and stained with all or some of the following conjugated monoclonal antibodies: CD122 (BV421, BD Biosciences, 562960), CD4 (APC-Cy7, Biolegend, 100413), CD3 (Brilliant Violet 510, Biolegend, 740113), FcγRIIB (Alexa Flour 488, Cell Signaling Technology, 54837), CD27 (Pacific Blue, Biolegend, 124218), CD138 (PE, Biolegend, 112503), CD19 (Brilliant Violet 480, Biolegend, 566107), Siglec-G (Alexa Flour 647, Biolegend, 563336), IgD (Brilliant Violet 605, Biolegend, 406527), IgM (PerCP/Cyanine 5.5, Biolegend, 406512), IgG (PE/Cyanine 7, Biolegend, 405315). Separate panels included I-Ad (PE, Invitrogen, MA5–17780), and H-2kd (PerCP Cyanine 5.5, Biolegend, 1147115). Surface marker antibodies were diluted to 1:200 (or per manufacturers specifications), and 100uL was added to samples and incubated in darkness for 30 minutes. Samples were washed three times with FACS buffer and finally resuspended in order to be analyzed via flow cytometry on Cytek Aurora Flow Cytometer (Cytek Biosciences). Gating strategies were controlled using fluorescent minus one (FMO) controls and universal negatives in the form of unstained cells, as well as cells stained with each isotype-controlled mAb. Samples were then analyzed on FlowJo software (Tree Star). Figures from flow cytometry are representative of the triplicate samples.

### Donor-Specific Antibody Detection

The presence of donor- specific antibodies (DSA) were tested for within the recipient’s sera, after its having been isolated from whole blood. Each DSA experiment contained donor splenocytes with an experimental group using recipient sera, a negative control group using the donor’s own sera, and a positive control group consisting of manufacturer antibody staining of the splenocytes. If previously frozen, all sera was thawed per thawing protocol over ice. Otherwise, 1:10 and 1:100 dilutions were prepared of the sera in FACs buffer. 100uL were then plated and incubated over the donor splenocytes (at 500,000 cells/well) for 30 minutes in darkness. Cells were washed with FACs buffer. IgG antibody dilution was prepared (Brilliant Violet 421, Jackson ImmunoResearch, 115–675-071) at 1:200 dilution, added to wells, and incubated for 30 minutes in darkness. Cells were washed twice using FACs buffer. Surface marker antibodies were then added to all samples; CD19 and CD3 (Biolegend, 566107, and 740113, respectively) were added to all wells, while MHC specific antibodies and isotypes (H2-Kd: Biolegend, 1147115; IA-d: Invitrogen, MA5–17780) were added to positive control samples. All wells were incubated for 30 minutes in darkness, and washed twice. Samples were then run on Cytek Aurora Flow Cytometer (Cytek Biosciences). Gating strategies and data analyses were performed as outlined in flow cytometry protocol.

### B Cell Isolation for Adoptive Transfer

Following normal cell dissociation protocol, B cells were isolated for using an EasySep B Cell Isolation Kit (Cat:19854) following the manufacturers protocol. In summary, the dissociated total cell mixture was exposed to an EasySep^™^ Mouse FcR Blocker antibody (Component: 18730), followed by the EasySep^™^ Mouse B Cell Isolation Cocktail (Component: 19854C). Both were incubated for 10 minutes at room temperature. 50uL of EasySep^™^ Streptavidin RapidSpheres^™^ 50001 (Component: 50001) per mL of solution, was added mixture following vortexing for 30 seconds. The solution was then incubated for 2.5 minutes at room temperature. The mixture was then placed in an EasyEights^™^ EasySep^™^ Magnet (Cat: 18103) for an additional 2.5 minutes until solution was clear, and all spheres had flushed to the magnet. Remaining B cells were then transferred into a 15mL tube, washed at 1600 RPM for 5 minutes, and a small portion (> a million cells) were set aside for flow cytometry staining and confirmation. The isolation was conducted in sterile conditions, with sterile tubes, under a fume hood for injection. The B cells were resuspended in RPMI at a concentration of 2×10^6^ per dose and injected intravenously through the tail vein into untreated recipients 2 days prior to kidney transplantation. Serum BUN and creatinine levels were regularly monitored to assess kidney function post-transplantation. Sera were also collected at this time for DSA. Pathological examination was performed on kidney allograft samples.

### Single cell RNA sequencing of kidney allografts and spleen

Isolated kidney or spleen cells were sorted for viable CD45^+^ cells via flow cytometry using an anti-CD45^+^ antibody (BioLegend,103114) and a fixable viability dye ([Invitrogen, L34976 A). These cells were used to construct single-cell RNA-seq libraries at Chromium 10x instrument using Chromium Next GEM Single Cell 3’ kit, which were sequenced in paired-end fashion^46^ on Illumina HiSeq 2500 instrument to the depth of approximately 100 million read pairs per sample.

### Bioinformatics analyses of scRNA-seq data of kidney allografts and spleen

The raw sequencing data underwent initial mapping and processing using the CellRanger package (Chromium Genomics). The resulting read counts were further analyzed using Seurat^47,48^ and Monocle^29–31^. This included filtering cells by the number of UMIs, mitochondrial content, and number of expressed genes, as well as further normalization and scaling of read counts. Datasets for various time points (1 week, 3 weeks, and 24 weeks for accepted recipient and 1 week for rejecting recipient) and biological replicates (N = 3 at 1 week, N = 5 at 3 weeks, and N = 3 at 24 weeks) were integrated, followed by principal component analysis (PCA), generation of Uniform Manifold Approximation and Projection (UMAP) plots, and cell clustering using Seurat functions with default parameters. All mouse replicates were integrated using Seurat after scRNAseq was performed. This created a data object for all pooled samples together. While each mouse was a separate sample, replicates in a single time point were pooled and compared across other time points as well. Annotation of cell types was performed manually by investigating canonical markers in literature. Gene expression analysis across time and cluster type was performed using Seurat software^47,48^ and Nebulosa packages^49^.

Integrated data from Seurat analysis was then transformed into a Monocle 3 object, including expression data and cell-level metadata using the as.cell_data_set function to allow trajectory analysis. This object underwent further analysis, including unsupervised clustering of cells using the cluster_cells function and updating the Monocle 3 object. Next, the Monocle 3 object underwent analysis using the learn_graph function to determine the biological program of gene expression for the scenario in question and, therefore, learned the trajectory of cells through this higher-dimensional space. Lastly, the order_cells function chooses root states using the interactive Monocle 3 online software and determines the pseudotime values based on the object produced using the learn_graph function. When this was complete, various qualitative graphs were produced to illustrate the trajectories of cell types in a larger population.

### scRNAseq data of KPC tumor

A mouse model of pancreatic ductal adenocarcinoma was employed that combined the tamoxifen-inducible pancreatic acinar cell-specific expression of the Cre recombinase (Ptf1a-CreER) with the expression of oncogenic Kras^G12D^ (LSL-Kras^G12D^) and heterozygous loss of Tp53^+/fl 50–52^. Mice were administered tamoxifen at 6 weeks of age and tumors were harvested for scRNA-seq analysis at 6 months of age. Approximately 50 mg of tumor was enzymatically disassociated in RPMI containing 0.1 mg/ml DNase I (Roche), 0.2 mg/ml Collagenase P (Roche), 0.1 mg/ml Dispase (Gibco-Thermo Fisher), and 2% fetal bovine serum (FBS). The cell suspension volumes were calculated for a target cell recovery of between 4000 to 8000 cells and loaded on the Chromium using 10x Genomics Chromium Single Cell 3’ Reagents v3 kit (10x Genomics) according to the manufacturer’s guidelines. Purified cDNAs were quantified using High Sensitivity D5000 ScreenTapes and Reagents on an Agilent Tapestation (Agilent). The final single cell 3’ libraries were quantified using an Agilent Tapestation using High Sensitivity D1000 ScreenTapes and Reagents. Libraries were loaded at 160 picomolar on an Illumina cBOT and sequenced on a HiSeq4000 for 28 base pairs on the first read, followed by an 8 base pair index read, and a 91 base pair second read, using 2 HiSeq4000 SBS kits, for 50 cycles. Illumina Real Time Analysis software was employed to generate sequence intensity files which were then demultiplexed and aligned to the human genome, version hg38, using the 10x Genomics CellRanger v3.0.1 software package.

### Evaluating B cell Signature enrichment together with SIGLEC10 expression in metastatic melanoma patients treated with anti-PD1

We analyzed publicly available transcriptomic data from Huang and colleagues^35^. We focused specifically on pre-treatment tumor samples from immunotherapy naïve patients with metastatic cutaneous melanoma treated with anti-PD1(Programmed cell death protein-1) monotherapy. Response was evaluated either by RECIST v1.1 where available, or by best overall response (BOR). Patients with a RECIST or BOR clinical response of complete or partial response (CR, PR) or stable disease (SD) with a progression-free survival (PFS) greater than 6 months were categorized as not progressive disease (notPD). This aggregated group was compared with patients with a RECIST or BOR response of progressive disease (PD). To maintain well-defined cohorts, patients with mixed response (MR) or non-evaluable response (NED) were excluded. From these cohorts, we estimated B cell enrichment using single-sample Gene Set Enrichment Analysis (ssGSEA) implemented in the GSVA R package (R version 4.2.0). The B cell signature gene list was obtained from www.Nanostring.com, defined by the expression of the following genes: BLK, CD19, MS4A1, TNFRSF17, FCRL2, FAM30A, PNOC, SPIB, and TCL1A. The ssGSEA scores were computed on normalized expression data to obtain a B cell enrichment score for each sample. B cell enrichment scores analysis compared samples in response group PD with those in notPD. The ssGSEA scores were then z-score transformed across samples and included in multivariate logistic regression models to evaluate their association with treatment outcome. Expression levels of individual genes, including SIGLEC10, were also assessed independently and in combination with the B cell signature.

### Bioinformatics analyses of scRNA-seq data of human melanoma datasets

Human melanoma cancer cell data sets included in GSE115978^34^ were obtained from the Gene Expression Omnibus (GEO). Cells from tumor samples were included based on the following exclusion criteria (1): cells with greater than 10% mitochondrial genes (2), cells expressing less than 200 genes or greater than 7,000 genes. Following quality control, read counts were normalized and scaled. Datasets were processed by PCA, followed by UMAP visualization and cell clustering using Seurat functions with default parameters. Annotation of cell types was performed manually by investigating canonical markers in literature. Gene expression analysis in T cell subsets was performed using Seurat software^47,48^, Nebulosa packages^49^, and Monocle 3 packages^29–31^.

### Statistics

Data is presented as mean ± standard deviation (SD) for technical replicates and mean ± standard error of the mean (SEM) for biological replicates. Variables among groups were compared using the 2-tailed Student's t-test for comparison of 2 conditions; 1-way ANOVA test for comparison of more than 2 conditions. A P value less than 0.05 was considered significant. Allograft survival curves were constructed by the Kaplan and Meier method, and comparisons were performed using the log-rank test. These analyses were performed with Prism v10.0 (GraphPad Software) and IBM SPSS statistic v28.0 (IBMcorp).

### Study approval

All research with animal models was subject to prior review and approval and conducted in compliance with institutional guidelines set forth by the Animal Care and Use Committees of the Massachusetts General Hospital.

## Supplementary Material

1

## Data Availability

Raw and processed scRNAseq data for accepted and rejecting kidney allografts have been deposited into the Gene Expression Omnibus (GSE252337) database. ECDI-SP induced tolerance model’s data sets, GSM4761001 and GSM4761004, contained in dataset GSE157292 were downloaded from the Gene Expression Omnibus (GEO). Other data are available upon reasonable request from the corresponding authors subject to institutional review and approval.
