# Supplementary material for: Defensive tolerance drives the reprogramming and dysfunction of infiltrating pathogenic B cells assuring the maintenance of tolerance
Source: Res Sq. 2025 Aug 18:rs.3.rs-7236564. Preprint. [Version 1] doi: 10.21203/rs.3.rs-7236564/v1 (PMC12393600; doi:10.21203/rs.3.rs-7236564/v1)
Supplement: 1 [file NIHPPRS7236564V1-supplement-1.pdf]

Supplemental Figure 1

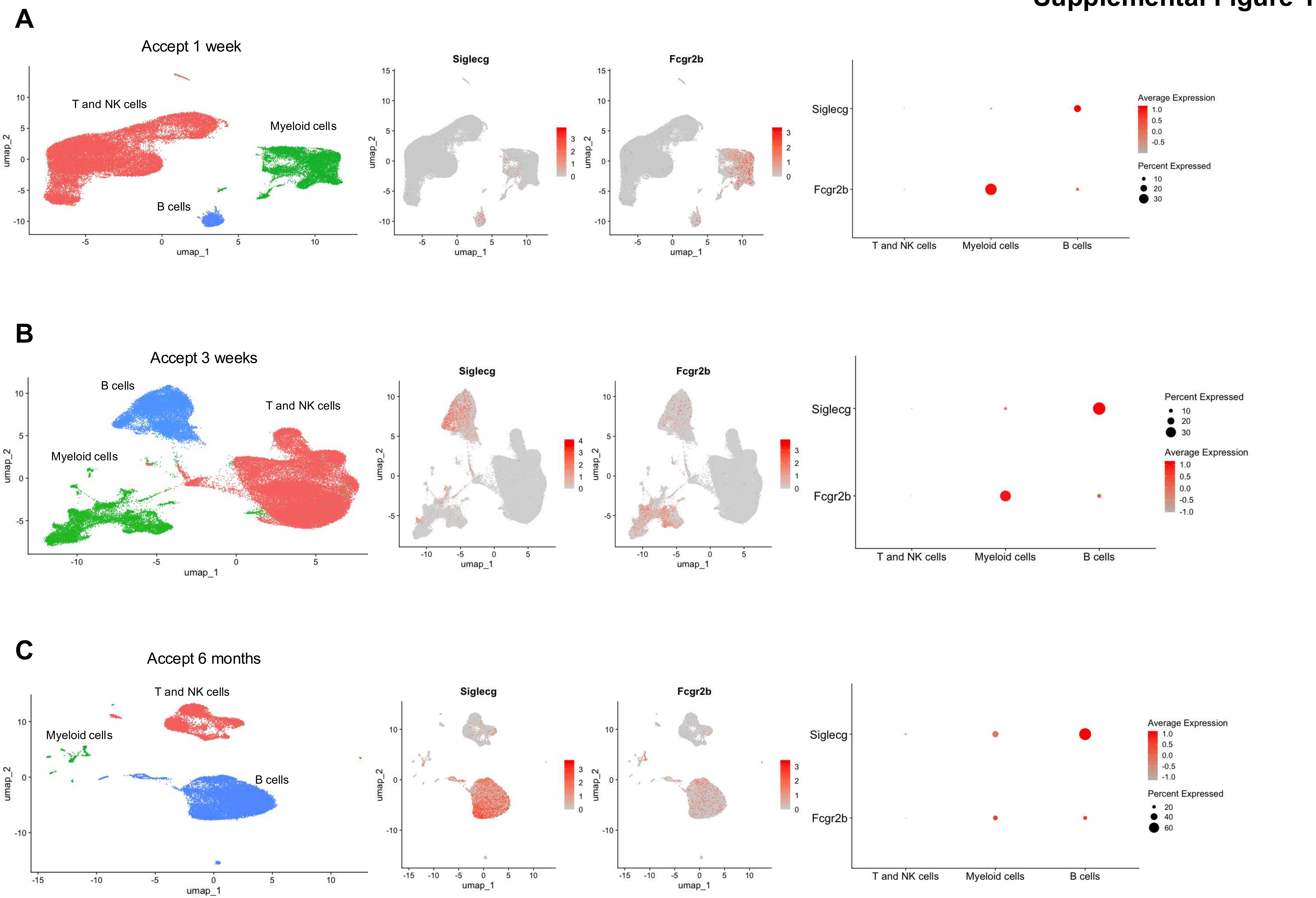

Supplemental Fig. 1. **Temporal intra-graft expression of *Fcgr2b* and *Siglecg* within the B and myeloid cell clusters in accepted kidney allografts.** (**A**) Expression of *Fcgr2b* and *Siglecg* at 1 week post-transplantation. From left to right, UMAP showing B cell, myeloid cell, T/NK cell clusters; feature plot and dot plot showing *Fcgr2b* and *Siglecg* expression. (**B**) Expression of *Fcgr2b* and *Siglecg* at 3 weeks post-transplantation. From left to right, UMAP showing B cell, myeloid cell, T/NK cell clusters; feature plot and dot plot showing *Fcgr2b* and *Siglecg* expression. (**C**) Expression of *Fcgr2b* and *Siglecg* at 6 months post-transplantation. From left to right, UMAP showing B cell, myeloid cell, T/NK cell clusters; feature plot and dot plot showing *Fcgr2b* and *Siglecg* expression.

Supplemental Figure 2

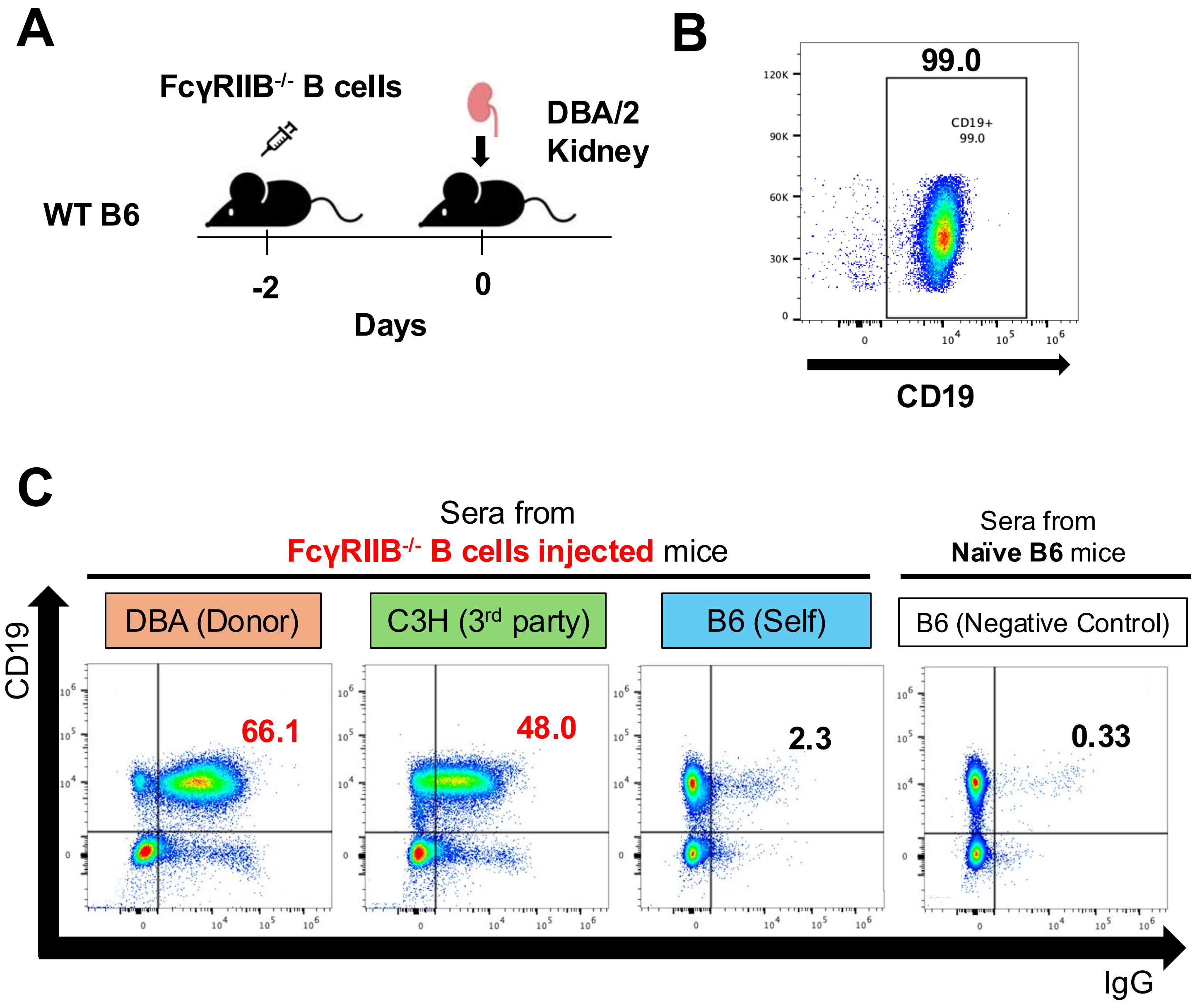

Supplemental Fig. 2. **Donor-specific antibody responses in recipients injected with FcγRIIB$^{-/-}$ B cells.** (**A**) Schematic showing the experimental design for FcγRIIB$^{-/-}$ B cells injection followed by kidney transplantation in a tolerance model (DBA/2 to wild-type B6 recipients). (**B**) Flow cytometric analysis of CD19^{+} cells following B cell isolation from the spleens of FcγRIIB$^{-/-}$ B6 mice. (**C**) Flow cytometric analysis of serum antibodies reactive to mouse spleen cells. Sera from recipients injected with FcγRIIB$^{-/-}$ B cells exhibited higher antibody reactivity against DBA/2 (donor, 66.1%) and C3H (third-party, 48.0%) cells, compared to B6 (self, 2.3%) and negative control (0.33%). N=3
